# New Prospects in the Electroanalysis of Heavy Metal Ions (Cd, Pb, Zn, Cu): Development and Application of Novel Electrode Surfaces

**DOI:** 10.3390/mps6040060

**Published:** 2023-06-26

**Authors:** Vasiliki Keramari, Sophia Karastogianni, Stella Girousi

**Affiliations:** Analytical Chemistry Laboratory, School of Chemistry, Faculty of Sciences, 54124 Thessaloniki, Greece; vasilikik20keramari@gmail.com (V.K.); skarastogianni@hotmail.com (S.K.)

**Keywords:** heavy metal ions, modified electrode, electroanalysis, nanoparticles

## Abstract

The detection of toxic heavy metal ions, especially cadmium (Cd), lead (Pb), zinc (Zn), and copper (Cu), is a global problem due to ongoing pollution incidents and continuous anthropogenic and industrial activities. Therefore, it is important to develop effective detection techniques to determine the levels of pollution from heavy metal ions in various media. Electrochemical techniques, more specifically voltammetry, due to its properties, is a promising method for the simultaneous detection of heavy metal ions. This review examines the current trends related to electrode formation and analysis techniques used. In addition, there is a reference to advanced detection methods based on the nanoparticles that have been developed so far, as well as formation with bismuth and the emerging technique of screen-printed electrodes. Finally, the advantages of using these methods are highlighted, while a discussion is presented on the benefits arising from nanotechnology, as it gives researchers new ideas for integrating these technologies into devices that can be used anywhere at any time. Reference is also made to the speciation of metals and how it affects their toxicity, as it is an important subject of research.

## 1. Introduction

Most heavy metal ions exist naturally in the environment; however, some come from anthropogenic sources, such as some industries, agriculture, the burning of fossil fuels, insecticides, car exhausts, and sewage. These heavy metal ions in large quantities can become hazardous to the biological system. For example, cadmium (Cd), lead (Pb), zinc (Zn), and copper (Cu) affect the environment due to their non-biodegradability and accumulated toxicity [1].

In the ground, all of the inorganic elements that are necessary and essential for the normal growth and development of plants exist. Even though some heavy metal ions, such as copper (Cu) and zinc (Zn) etc., are necessary for various enzymatic functions, an excessive concentration of heavy metal ions can cause serious problems [2,3,4], as they can become toxic and dangerous, with serious environmental implications. Toxic heavy metal ions vary in their nature and mode of accumulation, either in the soil or in plants. Some of the sources of heavy metal ions in the soil are fertilizers, pesticides, and sewage sludge [5].

Toxic metals such as cadmium (Cd) and lead (Pb), as well as many others, can easily end up in the higher members of the biological food chain and, therefore, in humans, causing serious diseases such as gastrointestinal tract (GIT) infections, cardiovascular problems, bone problems, and even cancer [6,7]. On the other hand, equally serious are the effects that heavy metal ions have on the environment, e.g., soil pollution, which is one of the most important problems for the planet. The term “soil pollution” refers to the concentration of polluting substances in soil in quantities that cause a change in the composition of the soil, resulting in disturbances in the ecosystem. 

In order to limit the negative effects that heavy metals ion have on both humans and the environment, it is necessary to accurately determine the concentrations of heavy metal ions in their sources of accumulation.

Over the years, various techniques have been established for the detection of heavy metal ions (HMIs), including inductively coupled plasma mass spectrometry (ICP-MS) [8], inductively coupled plasma optical emission spectrometry (ICP-OES) [9], inductively coupled plasma atomic emission spectrometry (ICP-AES) [10], flame atomic absorption spectrophotometry (FAAS) [11], and atomic absorption spectroscopy (AAS) [12], where sometimes the emphasis is on the parameters and sometimes on the choice of the analysis method. Although they provide high accuracy and sensitivity, spectrometric methods such as atomic absorption (AAS) and inductively coupled plasma mass spectrometry (ICP-MS) are accompanied by certain limitations, such as high costs and the fact that they are time-consuming and do not allow on-site measurements.

However, as already mentioned, researchers’ interest in recent years has focused on the identification of heavy metal ions with the help of various electrochemical methods, particularly voltammetry, as it is an easy, fast, and relatively inexpensive way to determine them compared to other analytical methods. Although previous techniques are very sensitive and selective, due to the limitations they cause, electrochemical methods such as voltammetry are preferred for the detection of heavy metal ions, which, in contrast to previous techniques, have the advantages of low cost, simplicity, ease of operation, fast analysis, portability, the ability to monitor environmental samples in the field, and high sensitivity and selectivity [13]. Electrochemical techniques, especially voltammetry, involve electroanalytical methods for the determination of one or more analytes by measuring the current as a function of potential. However, voltammetry is the only electrochemical method that has high sensitivity and can be applied for the on-site recognition and detection of heavy metal ions [14]. Voltammetric modifications of ESA (i.e., DPASV, SWASV, and AdSV), including two variants of stripping potentiometry (PSA and CCSA), have historically been widely recognized as powerful techniques for detecting heavy metal ions due to their remarkable sensitivity, which allows for the detection at trace and ultra-trace levels.

Furthermore, there is also electrochemical-stripping analysis (ESA).

In order to improve sensitivity and selectivity in the detection of heavy metal ions and to examine their toxicity on both the environment and human health, we combine electrochemical techniques with certain modifiers that facilitate the detection process. Due to their characteristic properties, electrode modifiers were used in combination with electroanalytical methods.

The need for alternative electrode modifiers rose as attention began to turn more and more towards green chemistry, which aims to reduce the use and production of hazardous substances such as mercury [15], and since certain European regulations prohibit the export and storage of metallic mercury. Since then, several metals have been tested for their ability to replace mercury (e.g., bismuth). In addition, different organic and inorganic membranes have been evaluated for their potential application in detecting Pb with ESA.

In recent years, nanoparticles (NPs) have emerged as a promising area of research, replacing various electrode-shaping media, thanks to their unique physicochemical properties that differ from their bulk counterparts. NPs are defined as particles with a dimension of at least 100 nm, and are generally classified into four categories, including metal and metal oxide, semiconductors, polymers, and lipids. They exhibit a high surface area to volume ratio, which enhances their reactivity and allows them to interact with biological systems and the environment in unique ways. The ability of NPs to cross biological membranes and barriers has attracted considerable attention in various fields, including medicine, environmental science, and engineering, and they have been widely used for heavy metal detection, such as that for Cd, Pb, Zn, and Cu, due to their unique optical, electrical, and magnetic properties.

This review mainly discusses the use of voltammetry in the simultaneous detection of the presence of two or more heavy metal ions in different media using modified electrodes and presents a comprehensive overview of the modifiers for various electrodes. This paper includes a historical review from the abolition of mercury to its replacement and the discovery of innovative nanoparticles and presents their applications in chemistry and the environment. The present review also aims to summarize the different types of nanoparticles, such as metallic, semiconducting, and carbon-based nanoparticles, and their application in various electroanalytical techniques, including voltammetry.

## 2. Electrodes

Initially, the measurement technique is selected, with the most used measurement techniques for detecting heavy metal ions being SW, SWASV, and DPASW. In the next step, the appropriate working electrode is selected, where carbonaceous electrodes are dominant. They appear to further improve the performance of the voltammetric methods, as they are flexible, offer a wide potential window, and have desirable conductive and surface properties that allow for the sensitive determination of analytes. The four most common are glassy carbon electrodes (GCE), graphite electrodes (GE), carbon paste electrodes (CPE), and screen-printed carbon electrodes (SPCE). These electrodes are widely used for the determination of heavy metal ion concentrations (Cd^2+^, Pb^2+^, and Zn^2+^), while some of the most common types of modified electrodes include, among others, nanoparticle-modified electrodes, chemically modified electrodes (using chemical modifiers such as bismuth (Bi) and, formerly, mercury (Hg)), carbon-based modified electrodes, and enzyme-modified electrodes.

**Figure 1:**
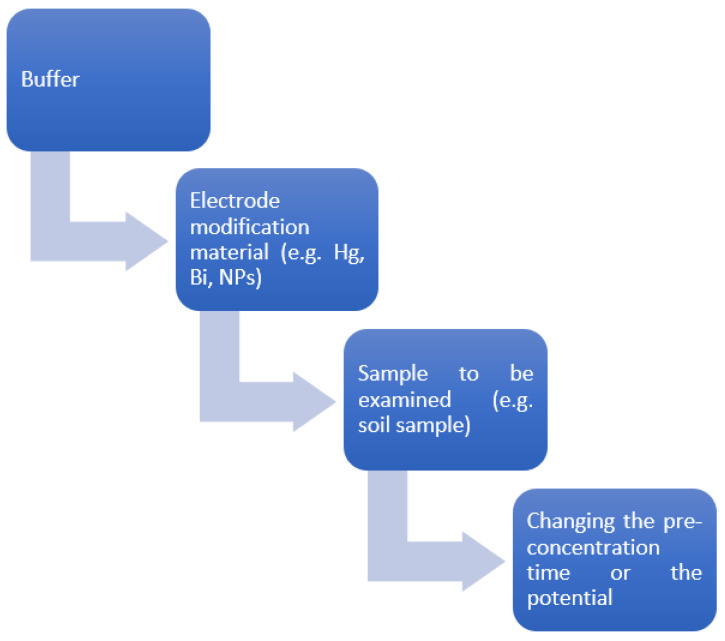
Sample preparation procedures for measurement.

Therefore, the appropriate electrode modifier is further investigated. In our case, we work with GCE, and our working electrode is modified with bismuth. In a recent study for the determination of lead, which is a heavy metal ion, and with GCE as the working electrode, modification was performed with BFS (blast furnace slag), which is an economically efficient process and a new material in the field of detection, with many promising results [16]. A typical electrochemical analytical system consists mainly of the following three parts: an electrochemical detection device, an electrochemical detection instrument, and an electrolyte. The electrochemical detection instrument usually consists of the following three electrodes: a working electrode (WE), a reference electrode (RE), and a counter electrode (CE). After modifying the surfaces of the Wes using different materials, they can be used for the specific detection of various types of metallic ions. A representative illustration of sample preparation is quoted schematically in Figure 1, where the main steps followed are shown. In some Wes, surface modification is necessary because the nature of the used electrode can greatly affect the sensitivity and the selectivity of the analytical procedure. For example, it is essential to polish the surface of a glassy carbon electrode (GCE) with a polishing cloth posing 0.1 mm and 0.005 mm alumina powder, inducing a mirror-like surface, which improves the analytical features of the detection procedure [14].

### 2.1. Glassy Carbon Electrode for HM Detection

One of the most common electrodes used for the detection and quantitative determination of heavy metals is glassy carbon electrodes (GCEs), which are produced by pyrolysis of polymeric resins. Some of their distinguishing characteristics are that they are easily based, non-reactive, resistant to high temperatures, and impermeable to gases and liquids, while simultaneously providing excellent analytical performance across a wide range of metals. With GCE, relatively low limits of detection (LOD) can be achieved [17], as demonstrated, for example, by Thanh et al., who modified the electrode surface with a Bi film and achieved LOD values of 1.07, 0.93, 0.65, and 0.94 ppb for Zn, Cd, Pb, and Cu, respectively, while maintaining high accuracy and repeatability within the measurements [18]. In another study, Hassan et al. determined Pb in the linear range of 10.0–120.0 μg L^−1^, Cd and Zn in the linear range of 0.0–50 μg L^−1^ (corresponding LOD values: 3.18 ng L^−1^, 0.107 μg L^−1^, and 0.037 μg L^−1^) in tap water simultaneously using SWASV with Hg–Bi film electrodeposition on GCE in the presence of poly(1,2-diaminoanthraquinone) [19].

### 2.2. Graphite Electrode for HM Detection

Graphite electrodes (GEs) are an effective tool in the electroanalytical determination of heavy metals, mainly preferred for the detection of Cd, Pb, and Cu. Their large surface areas, conductive properties, and low cost are characteristics that make GE an ideal choice for the electrochemical analysis of HMs [17]. For example, Donmez et al. were able to simultaneously determine Pb and Cd in water samples up to 0.46 and 1.11 μg L^−1^, respectively [20].

### 2.3. Carbon Paste Electrode for HM Detection

For the electroanalysis of heavy metals, carbon paste electrodes (CPEs) are commonly used due to the simplicity of preparation and formatting. To prepare a CPE, the carbonaceous material in the form of powder or powder in a high-viscosity oil, usually paraffin, mineral oil, or pyrite oil, needs to be added. They are often preferred because it is relatively easy to incorporate various substances, such as nanoparticles, waste materials, and various chemicals, into this mixture in order to enhance metal deposition on the surface [17]. In a study conducted by Zheng et al., the simultaneous detection of Cd and Pb was performed using CPE formatted with mesoporous alumina, yielding respective LOD results of 0.2 and 2.0 nM, respectivley [21]. However, with the appropriate formatters, suitable LOD levels can be achieved using CPE, at either lower or higher levels.

### 2.4. Screen-Printed Electrode (SPE) for HM Detection

Over the past two decades, significant effort has been made towards the development of more environmentally friendly and “green” electrode materials as substitutes, primarily for mercury electrodes. Screen-printed electrodes (SPEs), which are cost-effective electrochemical substrates, have undergone significant improvements in recent decades in terms of their form and printing materials. The main advantages of using SPEs are their flexibility and the fact that they can be used as disposable sensors (avoiding any potential contamination from previous measurements). Additionally, compared to other analytical methods, they are economically efficient, easily tunable, and suitable for integration into portable devices. SPEs may be the most suitable electrochemical sensors for in situ analyses due to their linear performance, low power requirements, fast response, high sensitivity, and ability to operate at room temperature [22,23].

SPEs are chosen as an economical substrate for electrochemical biosensor applications due to their small size, ease of mass production, and portability. To create an SPE, the electrodes must be prepared as pastes or inks so that they cannot be printed on the predetermined surface called the substrate, which can be made from ceramic or polymer material such as polyethylene terephthalate (PET) [17]. The surface of the SPE can be easily modified to suit multiple purposes related to different pollutants. There are many materials that can be used as the modifiers of SPEs for environmental analysis, including noble metals, inorganic nanoparticles, enzymes, and DNA sequences. For the determination of heavy metals such as Cd, Pb, Cu, and Zn, they can be modified with bismuth either in situ [24] or ex situ [25]. Bismuth is characterized as the most common modifier of SPEs for electroanalysis due to its good analytical performance and “environmentally friendly” characteristics [26]. Alternatively, bismuth can be deposited on SPEs in various forms, such as bismuth oxide [27,28,29], bismuth nanoparticles [30], and pre-deposited bismuth as a film [31]. Another material for the modification of SPEs is gold (Au), which is a valuable tool for the determination of Cu. Some researchers modified chitosan (CTS) on the surface of SPCEs for the simultaneous determination of Pb (II), Cu(II), Cd(II), and Hg(II), with a preconcentration time of only 30 s [32]. However, this specific material presents challenges in the determination of other heavy metals, as its cathodic overpotential for the reduction in hydrogen ions is low and the range of its cathodic polarization is limited. Therefore, Au electrodes are not particularly useful for the detection of metals with a more cathodic oxidative potential, such as Cd, Pb, and Zn [33]. They can also be modified with nanoparticles, such as carbon nanoparticles, for the determination of Cd^2+^, Pb^2+^, and Cu^2+^ ions [34,35], or chemically synthesized bismuth metallic nanoparticles that modify the screen-printed carbon electrodes that are used for the detection of Zn(II), Cd(II), and Pb(II) in liquid samples [30]. For example, McEleney et al., for the determination of Cd and Zn, modified the surface of their electrode, which was made of graphene oxide and graphitic carbon nitride, with bismuth and gallium by changing the pH within the same cell [35].

## 3. Modifiers

### 3.1. Mercury (Hg)-Based Electrode Variants and Bismuth Electrode Modifiers for HM Detection

To investigate the toxicity caused by heavy metal ions in soil, atmosphere, and consequently human health, and with the goal of limiting it, both techniques of analytical chemistry, specifically voltammetry in our case, and “supporting” means such as mercury (Hg) and bismuth (Bi), have been used, while in recent years nanoparticles (NPs) have been used.

For many years, mercury was used as the material for the modification of working electrodes used in trace element detection due to its high sensitivity, reproducibility, and renewability. Mercury-based electrodes have been widely used for several decades in the detection of heavy metal ions using electrochemical techniques, thanks to their large cathodic window, reproducibility, sensitivity, and low background [36,37,38].

However, mercury is a heavy metal that has become increasingly unpopular for use, due to its intense toxicity and bioaccumulation in many species [38,39,40,41,42]. The danger associated with mercury-based electrodes is in their use, handling, and disposal due to their toxicity. In addition, it has been repeatedly shown that the absorption of Hg harms human health, as it can lead to many serious problems, such as neurological consequences (as it penetrates the blood–brain barrier), memory loss, insomnia, neuromuscular changes, and various effects on the renal system.

Over the years, various materials, such as noble metals (Pt, Pd, Au, and Ag) and other metals (Ru, Cu, Co, Ni, Pb, Sb, Bi, and Al), have been proposed and tested to replace mercury in the electrode modification process [43,44]. Although it is a heavy metal ion, the metal that prevailed is bismuth, due to its low toxicity [38,39,41,45,46], as well as its similar electroanalytical properties to mercury, such as a wide potential window, simple preparation, partial insensitivity to dissolved oxygen, and ability to form alloys with different metals [38,39,40,47,48]. Bismuth is also environmentally friendly [49] and has mostly succeeded in replacing mercury, as the latter is quite toxic. Therefore, around 2000, electrodes modified with bismuth were introduced that were constructed from a layer of bismuth deposited on a suitable substrate [39,50] and represented a very attractive alternative solution to the commonly used mercury electrodes [45]. Many different materials have been used as electrode substrates, such as carbon, glassy carbon, carbon fibers, carbon paste, graphite, wax-impregnated graphite, gold, and platinum [33,34,35,36,37,38,39,40,50]. The current peaks obtained in the voltammograms when using bismuth electrodes tend to be sharp and well-defined [45], allowing for the reliable, fast, and economical recognition and quantification of metals present in the sample. Due to its characteristics, bismuth can be used as a film in electrodes, such as in glassy carbon electrodes (GCEs), and then can be used in various sample analyses (environmental, biological, etc.).

An example worth mentioning is the simultaneous detection of different heavy metals ions that are present in a sample, which is performed following the modification of vitreous carbon electrode with bismuth. Then various experimental parameters, such as potential and deposition time, are optimized, and finally, the appropriate voltammetric method, square wave voltammetry (SWV), is used [14].

Electrochemical detection focuses on developing new electrode materials with better properties compared to commercial electrodes. The performance of the voltammetric determination of heavy metal ions depends heavily on the properties of the working electrode. Working electrodes can be modified with different materials to allow for specific recognitions and concentrations of metal ions. Additionally, it has been reported that the deposition of metal membranes on nanocarbon materials can further improve the electrochemically active surface [15,51]. Among these, bismuth (Bi) film not only has low toxicity, high sensitivity, and a strong response signal, but it can also form binary or multiple-component alloys with heavy metal ions, which is a process that is analogical to the amalgamation with mercury, also enhancing the efficiency of the deposition at the surface (of either elemental mercury or bismuth).

One of the earliest applications of a bismuth-modified electrode was for the determination of lead in water samples using electrochemical stripping analysis (ESA), and, because it is considered one of the least toxic metals, it has subsequently been used for analyses in the medical and pharmaceutical sectors [52]. For approximately 20 years, bismuth-modified electrodes, which emerged as a replacement for toxic mercury, have found a wide range of environmental and clinical applications. Therefore, bismuth films are often combined with carbon materials for cooperative heavy metal detection. Hutton et al. [53] used a bismuth film for stripping measurements of cobalt and cadmium internal soil extracts. Recently, Bi-modified electrodes have also been successfully used in the electrochemical detection of nitrophenols, while bismuth oxides have been used in the detection of paracetamol.

### 3.2. Inorganic Materials as Electrode Modifiers for HM Detection

Another method for the detection of HMs is the surface modification of electrodes with inorganic materials, as this method can improve the sensitivity, stability, and selectivity of the electrode for HM ion detection. It has been found that inorganic nanoparticles modified on the electrode surface can adsorb more HM ions, thereby enhancing the specific surface area of the working electrode. They can also play a catalytic role in the deposition of HM ions on the electrode surface, thereby improving the electrochemical detection capability. However, a disadvantage of this is that inorganic nanoparticles are relatively expensive and challenging to produce on a large scale [54].

Some of the inorganic materials that have been successfully used for electrode modification and HM detection are metal and metal oxide nanoparticles, such as noble metal nanoparticles (e.g., AuNPs), bimetallic, and metal oxide nanoparticles. They have been employed to modify the electrode surface due to their favorable optical and electrical properties. They can be combined with other chemicals and biomolecules to construct various highly specialized electrochemical detection devices for HM ions. An example of this is the electrodeposition of AuNPs and Bi film on a screen-printed carbon electrode (SPCE) to obtain Bi/AuNP/SPCE, where the synergistic effect of the Bi membrane and AuNPs increased the surface area of the electrode, with good electrical conductivity. Using the differential-pulse anodic stripping voltammetry (DPASV) method, with detection limits of 50 ng/L (Zn^2+^), 20 ng/L (Pb^2+^), and 30 ng/L (Cu^2+^), the successful simultaneous detection of Zn^2+^, Pb^2+^, and Cu^2+^ in lake water was achieved [55].

### 3.3. Nanoparticles as Electrode Modifiers for HM Detection

As we have already mentioned, pollution from heavy metal ions is a significant issue, and, currently, the addition of NPs with electrochemical sensors has developed a significant and innovative analytical technique for the detection of heavy metal ions (HMs), as nanomaterials have been shown to offer remarkable properties as detection platforms. Nanomaterials could be considered as a promising tool for the scientific community to detect toxic heavy metal ions, due to their sensitivity and selectivity, fast response time, high sensitivity, and reproducibility, as well as the possibility of the simultaneous detection of HMs with very low detection and quantification limits [56]. Over time, many different modification techniques have been explored. Recent studies have shown that NP-modified electrodes can be very useful in electrochemical sensor technology if they are designed and constructed correctly [57]. Their surface area-to-volume ratio is high, and, in combination with the characteristics exhibited by NPs, such as those based on metals and metal oxides, polymers, and carbon, they can be beneficial for removing HMs from the environment [58].

Nanotechnology and nanoparticles (NPs) have transformed science and technology. Today, this field has advanced to such a degree that it allows the development of the production of nanoparticles using various physical, chemical, and even biological techniques. Among these techniques, the one that stands out and is preferred more in the industrial sector to produce nanoparticles is the biological method, due to its ease, the need for mild operating conditions, and the production of more environmentally friendly products and waste [59]. Most industries today exploit the chemical properties of nanoparticles, as they are unique compared to their counterparts in volume, which is determined by their size, shape, composition, and surface chemistry and can be adapted to various applications. Some of the most important chemical properties of nanoparticles are as follows [60]:

- The high surface-to-volume ratio: NPs have a high surface-to-volume ratio, which makes them extremely reactive. This property can be used in various applications, such as catalysis and sensors.
- Surface energy: The surface energy of NPs is high due to the presence of unsaturated surface atoms. This property affects the agglomeration, stability, and dispersion of NPs.
- Electromagnetic properties: NPs can exhibit unique electromagnetic properties due to their size, shape, and composition. For example, gold NPs exhibit localized surface plasmon resonance, which can be used for sensing and imaging applications.
- Surface chemistry: The surface chemistry of NPs can be tailored by modifying their surface functional groups, which can change their surface reactivity and chemical properties.
- Oxidation-reduction properties: NPs can exhibit unique oxidation-reduction properties, due to their small size and large surface area. This can be utilized in various applications, including energy storage and conversion.

The synthesis of NPs using the bio reduction method has drawn scientific interest, as it has managed to overcome the drawbacks of using conventional chemical methods, such as thermodynamic stability, monodispersity, and particle formation [61]. The biogenic synthesis of NPs presents some advantages over chemical synthesis, such as the absence of the need for high temperatures, toxic chemicals, pressure, energy, radiation processes, laser ablation, and ultraviolet and ultrasonic fields, as well as the fact that the biomolecules required for NP synthesis are abundant and easily accessible, such as the availability in marine sources [62]. On the other hand, NPs produced from the noble metal group, such as gold (Au) and silver (Ag), exhibit interesting chemical and electromagnetic properties, such as chemical stability, conductivity, and good optical properties, due to their ability to interact with electromagnetic radiation [63,64].

NPs, due to their large surface area, are excellent electron mediators. Therefore, NPs suitable electrode surface modifier the improvement of the analytical characteristics of electrodes. For instance, silicon (Si)- and carbon-based nanoparticles and have been successfully used as electrode modifiers. By using this kind of modification, the behavior of these NPs improves, and the constructed electrochemical sensors can measure the analytes in nanoscale. Thus, the use of NPs as electrode surface modifiers increases the active surface area, catalytic activity, conductivity and makes the response of the used electrodes more rapid. These redesigned sensors can also exhibit size-dependent characteristics and can have better functional units [65]. Currently, the addition of these NPs to electrochemical sensors has developed a significant analytical technique for detecting heavy metal ions (HMs).

Gold is excellent for the fabrication of nanomaterials because gold nanoparticles (AuNPs) are characterized as excellent templates for the development of cutting-edge chemical and biological sensors, thanks to their unique physical and chemical properties. AuNPs can be easily produced and made very stable [66]. They also have exceptional optical-electronic properties, and, with the right linkers, they offer a high surface-to-volume ratio and great biocompatibility. Furthermore, AuNPs can provide a versatile substrate for attaching a wide variety of chemical or biological moieties, allowing the selective capture and detection of small molecules and biological targets. It must be stressed that, when HMs are analyzed, particularly mercury and lead, different materials are incorporated with AuNPs, while the same materials can also be used for the detection of Cd (II) and Pb(II) [67]. According to the composition conditions, gold nanoparticles (AuNPs) appear in a variety of shapes, such as spherical, which is the most common shape used in the electrochemical detection of heavy metals, with sizes ranging from 4 to 298 nm. Different composition shapes of AuNPs were tested for the detection of Pb (II). According to the literature, for Pb (II) detection, Dutta et al. synthesized nano-stars, which were prepared by mixing an auric chloride solution with 4-(2-hydroxyethyl)-l-piperazineethanesulfonic acid (HEPES) without stirring or agitation, and boiling the nano-stars for 5 min resulted in spherical nanoparticles. The same process was later used for the synthesis of gold nano-stars for Cd (II) detection [68]. To evaluate the concentrations of cadmium in different water sources (such as lake, sewage, tap water, and groundwater), a glassy carbon electrode was modified with AuNPs, l-cysteine, and reduced graphene oxide, and, by applying square-wave voltammetry, the best performance for Cd (II) detection was achieved. The same electrode also exhibited the highest reported sensitivity for Pb (II) detection [69].

Other NPs, such as superparamagnetic Fe_3_O_4_@EDTA, have been developed for the simultaneous adsorption and removal of Zn(II), Pb(II), and Cd(II) from different environmental water and soil samples. For this method, which has been proven to be simple, fast, effective, sensitive with high removal yields, reproducible, and repeatable, electrodes modified with polymeric EDTA were used for the detection of various metallic ions at different pH values [70]. Furthermore, after the adsorption process, easy separation is provided only by the application of an external magnetic field. In conclusion, this method is an effective and less time-consuming technique for the simultaneous adsorption and removal of heavy metal ion targets in different environmental water and soil samples [71]. For the synthesis of Fe_3_O_4_@EDTA nanoparticles, the following procedure was performed: 15 mmol of FeCl_3_·6H_2_O and 7.5 mmol of FeCl_2_·4H_2_O were dissolved in 150 mL of deionized water under a nitrogen atmosphere at room temperature with vigorous stirring. Then, 50 mL of 25% NH_4_OH solution was added to the stirring mixture under intense mechanical stirring, adjusting the pH to 11, while simultaneously adding EDTA solution (3 mmol in 30 mL of water). The resulting black dispersion was continuously stirred for 1 h at room temperature and then refluxed for 2 h [71]. Finally, the resulting nanoparticles were isolated using a magnetic field; washed with water, ethanol, and diethyl ether; and dried. The simultaneous adsorption and removal of Zn(II), Pb(II), and Cd(II) in different environmental samples were successfully achieved with the aid of the superparamagnetic nano-adsorbent Fe_3_O_4_@EDTA.

Another category of NPs, AgNPs, which are used as electrode modifiers for the detection of heavy metal ions such as Cd(II) and Cu(II), have received significant attention due to some characteristics they exhibit, such as good electrical conductivity, high specific area, and an easy synthesis method [72,73]. It is supported by literature that, when the electrochemical technique is combined with nanomaterials, a very fast and efficient detection of heavy metal ions is obtained.

For the simultaneous determination of lead and cadmium, MnCo_2_O_4_ nanoparticles have been successfully used, which were morphed on a glassy carbon electrode. MnCo_2_O_4_ nanoparticles exhibit exceptional electrochemical properties, such as a fast current response, a low detection limit, and good selectivity, due to their unique structure [74]. The synthesis of MnCo_2_O_4_ nanoparticles was carried out using the citric gel combustion method as follows: solutions of manganese nitrate and cobalt nitrate were mixed in a molar ratio of 1:2, and citric acid was used as the fuel. The stoichiometric ratio of citric acid, according to the existing literature for the nitrate groups, was 1:3.6 [69], and the pH was adjusted to seven by adding an ammonium hydroxide solution. The mixture was then heated to approximately 80 °C in an open glass beaker under continuous stirring conditions (100 rpm) until a light-pink colloidal solution was formed, which was transformed into a gel and finally calcined at 450 °C for 2 h, resulting in the formation of black MnCo_2_O_4_ nanoparticles. For the measurement of Pb(II) and Cd(II) content in water samples, a glassy carbon electrode modified with MnCo_2_O_4_NPs was used. The electrode was immersed in a supporting electrolyte solution of H_2_SO_4_/KCl containing Pb(II) and Cd(II) during the pre-concentration stage. In this stage, the accumulation and reduction of metal ions to metal (M^2+^ to M^0^) occurred, while, in the deposition stage, the opposite process took place, i.e., the re-oxidation of metals (M^0^) and the stripping of metal ion species (M^2+^) from the solution. The electrochemical response was measured using the electrochemical technique of linear sweep anodic stripping voltammetry (LSASV) in the potential range of −1.0 to 0 V. The glassy carbon–MnCo_2_O_4_NPs electrode exhibited excellent electrochemical properties, such as a fast current response, a low detection limit, and good selectivity, due to its unique structure, as well as a satisfactory detection performance for Cd(II) and Cu(II) [74].

Lee et al. used tin nanoparticles (SnNPs) with reduced graphene oxide on a glassy carbon electrode to determine Cd(II), Pb(II), and Cu(II) [75]. For the simultaneous detection of Cd(II), Pb(II), and Cu(II) ions, G-Sn/GCS electrodes were used, which were derived when reduced graphene oxide (RGO) was activated with tin nanoparticles (SnNPs) and cast onto glassy carbon sheets (GCS), followed by electrochemical reduction. The results showed that the G-Sn/GCS electrodes exhibited good stability, high sensitivity, and good repeatability in heavy metal detection.

Bismuth nanoparticles (BiNPs) have attracted interest as pre-concentrators for the detection of heavy metals such as cadmium and lead ions, while they are also used as working electrode modifiers in stripping electrochemical analysis. Among the various reported methods for the synthesis of BiNPs, we have focused on the typical polyol method, which is widely used for these types of metallic and semimetallic nanoparticles [76]. Several techniques based on bismuth sulfide in combination with different working electrodes have been tested for the detection of heavy metals. Using square-wave anodic stripping voltammetry and a bismuth-nanodust-modified electrode, Lee et al. succeeded in detecting zinc, cadmium, and lead ions, followed by the preparation of spherical bismuth with different particle size distributions in order to investigate their effect on the sensitivity and detection limits of the detected metals. It was observed that, as the particle size decreased (from 406 to 166 nm), both the sensitivity and the detection limit improved [77]. On the other hand, Rico et al. [34] used the method of Lee et al. to detect heavy metals by forming a printed carbon electrode. The optimization of the method included accumulation configuration, resulting in better detection limits in the flow cells for Zn(II), Cd(II), and Pb(II) than in the batch cells. In another study for the determination of mercury, cadmium, lead, and copper ions using differential-pulse anodic stripping voltammetry, Sahoo et al. formed a graphene-oxide- and bismuth-nanoparticle-modified carbon paste electrode, with particle sizes ranging from 40 to 100 nm. A glassy carbon electrode was modified with a micro-nanoparticle/bismuth membrane by Saturno et al. for the determination of cadmium and lead using differential-pulse voltammetry. However, in almost all of these cases, the problem of copper (II) interference at high concentrations arose, which was addressed most of the time [67].

The application of palladium nanoparticles (Pd NPs) for the detection of heavy metals has been tested by a few research groups. However, they all synthesized porous activated carbon (PAC), followed by decorating PAC with palladium nanoparticles using a single-step thermal reduction method (with slightly different conditions). Zhang et al. used spherical Pd NPs, with a size range of 20–30 nm, for the individual and simultaneous determination of Cd(II), Pb(II), and Cu(II) through SWASV (square-wave anodic stripping voltammetry). The obtained detection limits for Cd(II), Pb(II), and Cu(II) were found to be lower in the individual determinations compared to the simultaneous determinations. The technique was successfully tested in practical water samples, although the nature of the water was not specified [78]. Summary of NPs-assisted detection of heavy metals ions is shown in Table 1.

As can be seen, in addition to their electrocatalytic properties, these nanomaterial-based electrodes have the advantages of low cost, high sensitivity, and convenient functionality, making them highly promising for practical applications in heavy metal detection. However, further research is required in order to overcome potential issues and improve the stability and selectivity of these sensors.

### 3.4. Ion-Imprinted Polymers as Electrode Modifiers for HM Detection

The determination of heavy metals in water and food intended for human consumption has led to an alternative method based on the modification of a working electrode with ion-imprinted polymers (IIPs). By using IIPs immobilized on a carbon paste electrode (CPE), the determination of both cadmium and lead ions can be achieved. The base of an IIP-modified CPE (CPEs-IIP) is usually formed by modifying a binary mixture, to which ingredients such as imprinted polymers are added or incorporated. The quantity can range from 10% to 30% of the composite mass, allowing for several recognition sites on the electrode surface, which correlates with the current intensity received. Additionally, IIPs can also be immobilized on a glassy carbon electrode (GCE) surface to detect cadmium, lead, cadmium, and pseudo silver ions in drinking water and food samples. In recent years, there has been significant interest in the CPEs-IIP technique, as it represents a simple and cost-effective method for the detection and analysis of heavy metal ions (Cd^2+^ and Pb^2+^) in both drinking water and food. This makes it a highly promising technique for improving everyday life [82].

### 3.5. Speciation of HMs

The speciation of chemical heavy metals is an important factor that alters the toxicity of heavy metals. The potential mobility, bioavailability, and environmental behavior of heavy metals depend largely on their specific chemical forms and existing conditions. Depending on the existing environmental conditions, various types of metal can exist as metals and metalloids, which can be present as hydroxides, organometallic compounds, biomolecules, and other forms, such as inorganic ions in the form of cations (e.g., Cd(II), Pb(II)), or anions (e.g., As(III) and As(V)). The determination of these molecular species is called metal speciation. Considering the toxicity and the bioavailability of heavy metals, their speciation is often more significant than determining total HMs. However, there are few publications that address the analysis and determination of different forms of a specific heavy metal, despite there being significant progress in the development of fluorescence detectors for detecting the total concentration of HMs [76].

Most of the current notification analysis methods for HMs combine separation techniques, such as gas chromatography, high-performance liquid chromatography, and so on, as well as detection techniques such as atomic absorption spectroscopy, atomic emission spectroscopy, and inductively coupled plasma mass spectrometry [83]. Although these methods have many advantages, a significant limitation is their requirements for a series of complex pre-processing steps, which are time-consuming and laborious. Electrochemical techniques have been characterized as the easiest, fastest, and most economical for species analysis. Van den Berg implemented the speciation analysis of different metallic elements such as iron, molybdenum, copper, and so on, using electrochemical methods [84,85,86]. ASV has been successfully adopted as the most common method for the analysis of metallic species, especially for unstable species [83]. Although voltammetric methods yield excellent results for the analysis of the speciation of many metal ions, there are certain issues and limitations that need to be addressed promptly, such as the relationship between bioavailability and metal speciation, which remains ambiguous and requires further study [87].

One example where speciation plays a crucial role is the exposure of plants to heavy metals through the aqueous phase of the soil, the soil solution, which contains heavy metals in various forms, such as free metal ions, simple inorganic complexes, and complexes with organic ligands. The composition of the soil solution can be influenced by the properties of the plants, as well as the soil itself. Environmental monitoring and the speciation of heavy metals (HMs) in soil solutions are highly important for ecological assessments and for understanding the plant–soil relationship [88]. Equally important is understanding the relationship between bioavailability and trace element speciation in natural aquatic environments [87].

## 4. Conclusions

Given that the global environmental burden of heavy metal ions and the associated impact on health and the environment are increasing, the interest in improving the quality of life and reducing their effects is ongoing. This review provides a general discussion on the field of electrochemical detection of HMs using bismuth-modified electrodes, which have replaced toxic mercury-modified electrodes, as well as nanomaterials as a more modern form of electrode modification, and their use in voltammetric experiments.

Certain materials, such as bismuth, have been distinguished for their ease of use, which is why researchers prefer them. The selection of suitable electrode modification materials is very important, as they improve the electrochemical properties of the electrode, increase its effective surface area for the transfer of the electrochemical signal, and produce detectable signals that are suitable for the indirect detection of HMIs. For the detection of HMs, voltammetric methods have been distinguished as the most powerful and sensitive and least time consuming.

In conclusion, the various nanoparticles that have been tested for the detection of heavy metal ions have shown significant results, due to the advantage of their large surface area compared to their size, as well as their electrocatalytic properties.

The purpose of the review was also to draw the attention of researchers working in electrochemistry, to develop new, improved morphology-controlled electrodes for the simultaneous detection of HMs at very low permissible limits (ppm, ppb), and, thus, reduce the quantity and toxicological burden of HMs in the environment [89].

## Figures and Tables

**Table 1:** Summary of NP-assisted detection of heavy metal ions.

Nanoparticles (NPs)	HM Detection	Ref.
AuNPs	Cd(II) and Pb(II)	[67,79]
Fe_3_O_4_@EDTA-NPs	Cd(II), Pb(II), and Zn(II)	[70,80,81]
AgNPs	Cd(II) and Cu(II)	[72,73]
MnCo_2_O_4_NPs	Cd(II) and Pb(II)	[74]
SnNPs	Cd(II), Pb(II), and Cu(II)	[75]
BiNPs	Cd(II) and Pb(II)	[76]
PdNPs	Cd(II), Pb(II), and Cu(II)	[67,78]

## Data Availability

Not applicable.

## Abbreviations

HMs, Heavy metal ions; ICP-MS, Inductively coupled plasma mass spectrometry; FAAS, Flame atomic absorption spectrometry; AAS, Atomic absorption spectrometry; ICP-OES, Inductively coupled plasma optical emission spectroscopy; ICP-AES, Inductively coupled plasma atomic emission spectroscopy; LSV, Linear sweep voltammetry; WE, Working electrode; ESA, Electrochemical-stripping analysis; RE, Reference electrode; CE, Counter electrode; EDTA, Ethylenediamine tetraacetic acid; SPEs, Screen-printed electrodes; CTS, Chitosan; AgNPs, Silver nanoparticle; AuNPs, Gold nanoparticles; NPs, Nanoparticles; SWASV, Square-wave anodic stripping voltammetry; GE, Graphite electrode; CPE, Carbon paste electrode; SPCE, Screen-printed carbon electrode; BFs, Blast furnace slag; Hg, Mercury; Bi, Bismuth; Cd, Cadmium; Pb, Lead; Zn, Zinc; Cu, Copper; Si, Silica; CV, Cyclic voltammetry; DPV, Different pulse voltammetry; GCE, Glassy carbon electrode; SWV, Square-wave voltammetry.
